# The Antimicrobial Peptide, Bactenecin 5, Supports Cell-Mediated but Not Humoral Immunity in the Context of a Mycobacterial Antigen Vaccine Model

**DOI:** 10.3390/antibiotics9120926

**Published:** 2020-12-19

**Authors:** Tulika Munshi, Adam Sparrow, Brendan W. Wren, Rajko Reljic, Samuel J. Willcocks

**Affiliations:** 1 St. George’s Medical School, University of London, London SW17 0RE, UK; tmunshi@sgul.ac.uk (T.M.); p1106405@sgul.ac.uk (A.S.); 2 The London School of Hygiene and Tropical Medicine, London WC1E 7HT, UK; brendan.wren@lshtm.ac.uk

**Keywords:** bactenecin, antimicrobial peptide, immunity, vaccine, mycobacteria, adjuvant

## Abstract

Bactenecin (Bac) 5 is a bovine antimicrobial peptide (AMP) capable of killing some species of bacteria through the inhibition of protein synthesis. Bac5 and other AMPs have also been shown to have chemotactic properties and can induce inflammatory cytokine expression by innate immune cells. Recently, AMPs have begun to be investigated for their potential use as novel vaccine adjuvants. In the current work, we characterise the functionality of Bac5 in vitro using murine macrophage-like cells, ex vivo using human tonsil tissue and in vivo using a murine model of vaccination. We report the effects of the peptide in isolation and in the context of co-presentation with mycobacterial antigen and whole, inert *Bacillus subtilis* spore antigens. We find that Bac5 can trigger the release of nitric oxide from murine macrophages and upregulate surface marker expression including CD86, MHC-I and MHC-II, in the absence of additional agonists. When coupled with mycobacterial Ag85 and *B. subtilis* spores, Bac5 also enhanced IFNγ secretion. We provide evidence that *B. subtilis* spores, but not the Bac5 peptide, act as strong adjuvants in promoting antigen-specific immunoglobulin production in Ag85B-vaccinated mice. Our findings suggest that Bac5 is an important regulator of the early cell-mediated host immune response.

## 1. Introduction

Antimicrobial peptides (AMPs) are conserved components of innate immunity that can be found across the taxonomic kingdoms [1]. They are comprised of varied sequences and secondary structures, but most are shorter than 100 amino-acids in length and carry a positive charge, facilitating electrostatic interaction with bacterial membranes. Some AMPs are modified by glycosylation, further diversifying their structure and function [2]. Bactenecin (Bac) family peptides are well conserved among ruminants [3], and are unusual in that they possess several repeating proline units in their sequence. Bac5, of which neutrophils are the major source in cattle, consists of a 43 amino-acid sequence in its cleaved, mature form, which is particularly highly expressed in milk, suggesting an important role in the prevention of mastitis [4].

Unlike many other AMPs that lyse bacteria through assembly in the inner-membrane [5], proline-rich AMPs can kill bacteria via inhibition of protein synthesis in the cytosol [6]. Transport appears to be mediated by a combination of endocytosis in eukaryotic cells [7], and inner-membrane transporter proteins in prokaryotes [6].

We and others have shown that the native Bac5 sequence exhibits species-restricted antimicrobial activity [6,8]. However, Bac sequences in general have also been the subject of rational design and synthesis to derive modified peptides that have novel properties [9]. For example, Bac family-derived, proline-rich peptide sequences have been explored as a mechanism for the delivery of noncovalently linked proteins into target cells [10].

AMPs represent potential alternatives to the use of antibiotics in human medicine and in farming, which may be drivers of antimicrobial resistance [11]. Innovative applications for use in animals include dietary supplements that stimulate protective AMP responses; in ovo delivery of peptides in poultry and fish to reduce infection prophylactically; and use as a new class of adjuvant for vaccines (reviewed by van Dijk et al. [12]). Exemplifying the latter, the bovine peptide, indolicidin, when co-administered with either ovalbumin [13] or hen-egg lysozyme [14] induces a T_H_2-bias that supports IgG production and improves IFNγ response upon restimulation.

We previously published findings that adsorption of early- and late-stage mycobacterial antigens (Ag85 and ACR, respectively) to *Bacillus subtilis* spores, combined with a fusion protein (heparin binding domain only protein) directs their trafficking to lung epithelial cells and supports the generation of mucosal immunity in a murine model when used as a booster for the Bacillus Calmette-Guérin (BCG) vaccine [15]. Novel vaccines and adjuvants are a current imperative to reduce the global burden of tuberculosis, the world’s leading cause of mortality by an infectious agent, due to the limited efficacy of the current BCG vaccine [16].

The most widely used types of adjuvant are aluminium salts. Alum-compounds tend to elicit a T_H_2 type immune outcome with associated cytokines such as IL-4 and IL-10 [17]. Broadly, T_H_2 bias is associated with antibody production, whereas T_H_1 bias is linked with the activation of cell-mediated killing effectors and associated cytokines such as IFNγ. Arguably, a T_H_1 response may be particularly important in TB vaccine/adjuvant strategies, and several approaches utilise toll-like receptor (TLR) agonists to achieve this outcome (reviewed by Stewart et al. [18]). Suitable correlates of protection for *Mycobacterium tuberculosis* (MTB) vaccines are the subject of much debate and ongoing research [19,20]. Alum-compounds also trigger inflammasome activation and the release of IL-1b, which contributes to dendritic cell maturation [21]. We previously showed that Bac5 was also capable of upregulating IL-1b in human macrophage-like cells [8].

There are, currently, several novel subunit vaccines in development for the next generation of tuberculosis vaccines [18]. Subunit vaccines typically lack the inherent antigenicity of live-attenuated, whole-cell vaccines, and alum-compounds are not ideally suited for use with peptide subunit-based vaccines because they contribute to their accelerated denaturation [17,22]. Alternative adjuvants, therefore, should be considered to gain maximal benefit from these promising new candidates.

In our previous work, we identified that Bac5 was a potent chemokine for macrophages in vivo, and was able to activate them in vitro [8]. We therefore hypothesised that this peptide may support antigen presentation to T-cells, potentially improving the efficiency of the host response to vaccination. We are also interested in the wider role of Bac5 in the immune response to infection, such as whether it is explicitly an innate immune effector, or if it also modulates adaptive immune responses in vivo. These questions cannot be answered using the embryonic zebrafish (*Danio rerio*) model we have used previously [8], since they lack T- and B-cells. We therefore sought to examine the functionality of Bac5 in the context of a human ex vivo tonsil tissue model and in a murine model of vaccination [15].

## 2. Results

### 2.1. Bac5 Stimulates Nitric Oxide Production in Murine Alveolar Macrophages and Alters Host Cell-Surface Marker Expression

**Figure 1:**
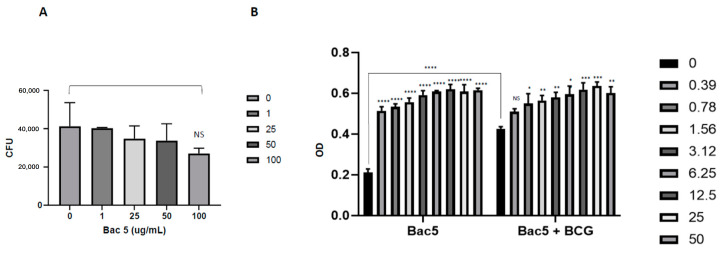
Activation of nitric oxide production from murine alveolar macrophage(MH-S) macrophage-like cells by Bac5 and effect on intracellular-resident MTB. MH-S cells were infected with MTB H37Rv at an MOI of 2 for 4 h in wells containing a titration of Bac5 peptide (0–100 µg/mL). This was followed by a wash step and addition of amikacin (200 µg/mL) to kill extracellular mycobacteria for 2 h prior to cell lysis and enumeration of viable bacteria by colony forming unit assay (**A**). Nitric oxide production by MH-S cells induced by Bac5 (0–50 µg/mL), or Bac5 with *M. bovis* BCG (MOI of 1) was assessed by Griess assay following 18 h coincubation of cells with stimuli (**B**). Absorbance was recorded at 560 nm using a spectrophotometer and two-way ANOVA performed to determine statistical significance (**** = *p* < 0.0001, *** = *p* < 0.001, ** = *p* < 0.01, * = *p* < 0.1, NS = No Significance) from triplicate technical repeats.

In our previous work, we demonstrated that Bac5 is capable of activating murine macrophage-like cells in vitro, and also had direct antimicrobial activity against some species of mycobacteria. In the current work, we studied the effect of preincubation with the peptide on the ability of murine alveolar macrophages (MH-S cells) to control *M. tuberculosis* infection. Although we observed a titratable effect, whereby the maximal dose of Bac5 (100 µg mL^−1^) reduced total intracellular bacterial burden, this was not statistically significant (Figure 1A). We confirmed that there was no cytotoxicity towards either MH-S cells or red blood cells caused by the peptide, and that Bac5 readily associated with MH-S cells (Figure S1). Consistent with our previous data using murine cell lines, we were able to confirm activation of MH-S cells via the production of nitric oxide (NO), detected by measuring nitrite in the culture supernatant, even when treated with low concentrations of Bac5 (<40 ng mL^−1^) (Figure 1B). Interestingly, this stimulatory effect of Bac5 was not enhanced further by the addition of live *M. bovis* BCG, which was able to independently stimulate NO production.

**Figure 2:**
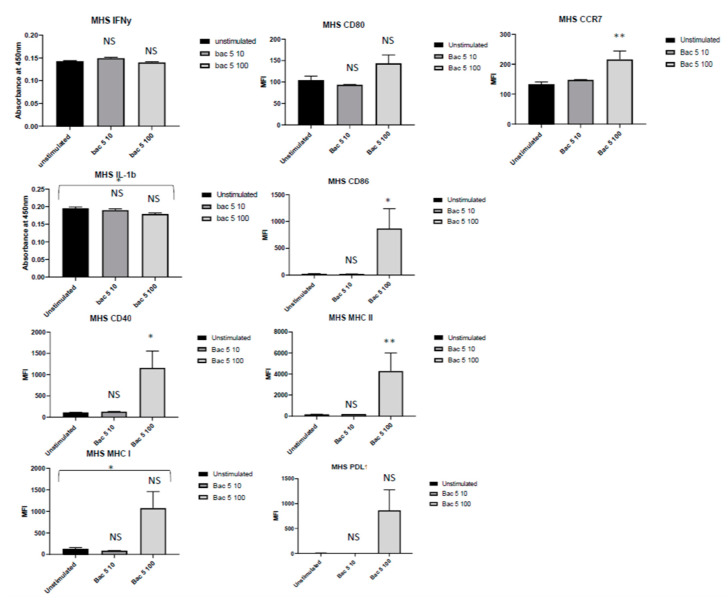
Upregulation of cell surface markers on MH-S Cells by Bac5. 1 × 10^6^ cells were incubated with either 10 or 100 ug/mL Bac5 peptide at 37 °C for 72 h. In all conditions, cells were initially stained with eFluor780 viability dye before subsequent immunostaining with fluorescently labelled antimurine target-specific IgG antibody as described. Mean fluorescent intensity (MFI) of at least 30,000 events was assessed by flow cytometry on viable cells using a Beckton Dickinson machine with FlowJo v.10.6.2. The data displayed is a representative figure from three independent repeats. One-way ANOVA with Kurskal-Wallis test for multiple comparisons was performed for each condition (** = *p* < 0.01, * = *p* < 0.1, NS = No Significance).

We furthermore analysed the expression of cell-surface markers on MH-S cells after treatment with Bac5 (Figure 2). We found that the peptide significantly increased the expression of CD86; CD40; MHC II and CCR7 in a dose-dependent manner. Expression of CD80 and MHC I was also significantly affected by Bac5 treatment according to one-way ANOVA statistical analysis, but a dose-effect was not present according to Dunnet multiple comparisons test.

### 2.2. Bac5 Stimulates IFNγ Release in Human Tonsil Tissue in Response to B. subtilis Spores with Ag85

**Figure 3:**
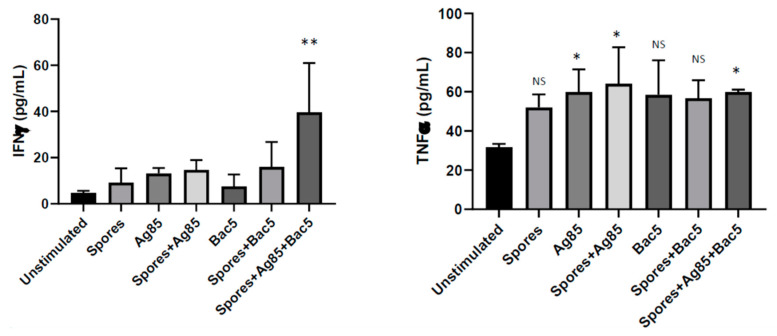
Cytokine Release by Stimulated Human Tonsil Cells. IFNγ and TNFα were quantified by ELISA from the cell culture supernatant of cells stimulated with either Bac 5 (10 ng/mL), *B. subtilis* spores (100:1 ratio to cells), Ag85B (5 μg/mL), or combinations as described. Absorbance was recorded at 450 nm and one-way ANOVA performed to establish statistical significance of each condition versus the unstimulated control (** = *p* < 0.01, * = *p* < 0.1, NS = No Significance) from triplicate technical repeats.

Having established an independent effect of Bac5, we next examined its activity in the context of additional pathogen associated molecular patterns (PAMP) through adsorption onto inert *B. subtilis* spores coated with mycobacterial Ag85 antigen. We utilised this approach as it has previously been validated as having adjuvant properties in support of BCG vaccination in murine models [15]. We confirmed binding of Bac5 to both live and dead spore surfaces through identification of FITC-labelled peptide using flow cytometry (Figure S2) and found up to 80% binding on 1 × 10^9^ spores using 25 µg mL^−1^ Bac5. When ex vivo isolated human tonsils were stimulated with Bac5 in combination with spore-Ag85, a significant increase in IFNγ was observed (Figure 3). This was only apparent when spore-Ag85-Bac5 were used in combination, not when the tissue was individually stimulated. Ag85 was able to induce TNFα secretion in tonsil cells alone, or in combination with either spores or spores plus Bac5. Unlike IFNγ, there was no significant additive effect of Bac5 on the amount of TNFα generated from Ag85 stimulation alone.

**Figure 4:**
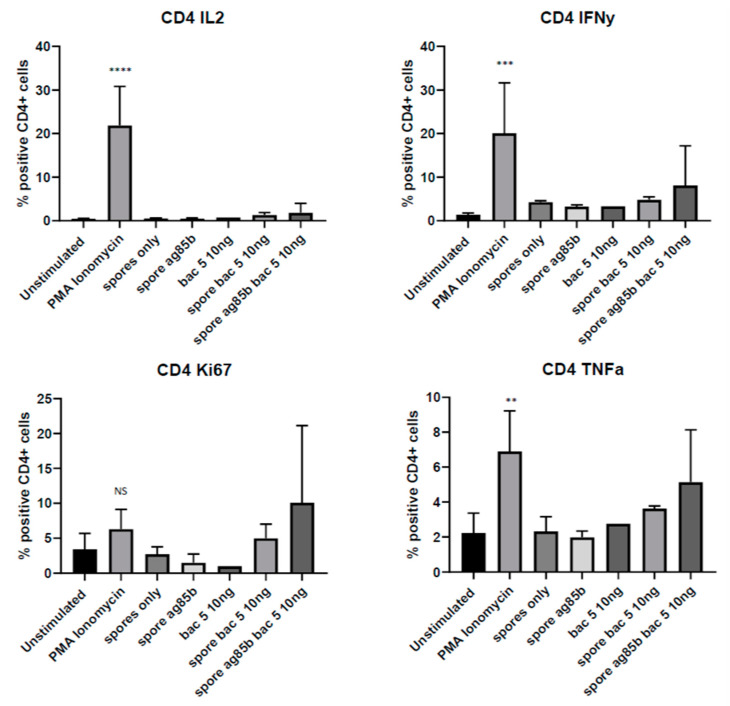
Intracellular Cytokine Production and Proliferation of Stimulated CD4^+^ Tonsil Mononuclear Cells. Human tonsil mononuclear cells were prepared as described and stimulated with different adjuvant combinations including *B. subtilis* spores (at a ratio of 100:1 to tonsil cells); Ag85B (5 µg/mL) and/or a titration of Bac5 peptide. Phosphomolybdic acid (PMA) ionomycin was used as a positive control. Cells were permeabilised with 0.5% saponin and intracellular cytokines stained using directly labeled target-specific IgG antibody. Fluorescence intensity was established on viable cells using flow cytometry. The percentage of CD4^+^ cells colocalising with positive fluorescence from either IL-2^+^, IFNγ^+^, Ki67^+^ or TNFα^+^ positive populations are displayed. Statistical analysis was performed using one-way ANOVA (**** = *p* < 0.0001, *** = *p* < 0.001, ** = *p* < 0.01, NS = No Significance).

**Figure 5:**
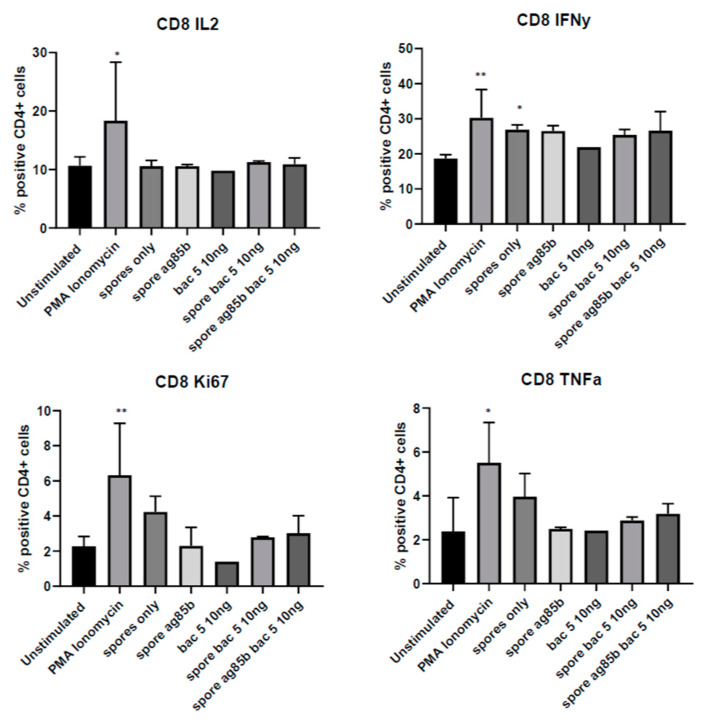
Intracellular Cytokine Production and Proliferation of Stimulated CD8^+^ Tonsil Mononuclear Cells. Human tonsil mononuclear cells were prepared as described and stimulated with different adjuvant combinations including *B. subtilis* spores (at a ratio of 100:1 to tonsil cells); Ag85B (5 µg/mL) and/or a titration of Bac5 peptide. PMA ionomycin was used as a positive control. Cells were permeabilised with 0.5% saponin and intracellular cytokines stained using directly labeled target-specific IgG antibody. Fluorescence intensity was established on viable cells using flow cytometry. The percentage of CD8^+^ cells colocalising with positive fluorescence from either IL-2^+^, IFNγ^+^, Ki67^+^ or TNFα^+^ positive populations are displayed. Statistical analysis was performed using one-way ANOVA (** = *p* < 0.01, * = *p* < 0.1).

Since tonsils represent a complex tissue comprised of a variety of cell types, including lymphocytes and epithelial cells, we used flow cytometry to isolate the effect of Bac5 specifically on the CD4^+^ and CD8^+^ T-cells sub-populations. Bac5 did not induce IL-12, Ki67, IFNγ or TNFα upregulation in either of the T-cell subsets, alone, or in combination with spores-Ag85 (Figure 4 and Figure 5). While a consistent trend that addition of Bac5 to spores-Ag85 resulted in a greater CD4^+^ cell activation, this effect was not statistically significant. The use of more spores or a greater sample size may thus be required.

### 2.3. B. subtilis Spores, But Not Bac5 Peptide, Increase Antigen-Specific IgG Titres in Response to Vaccination with Ag85B

**Figure 6:**
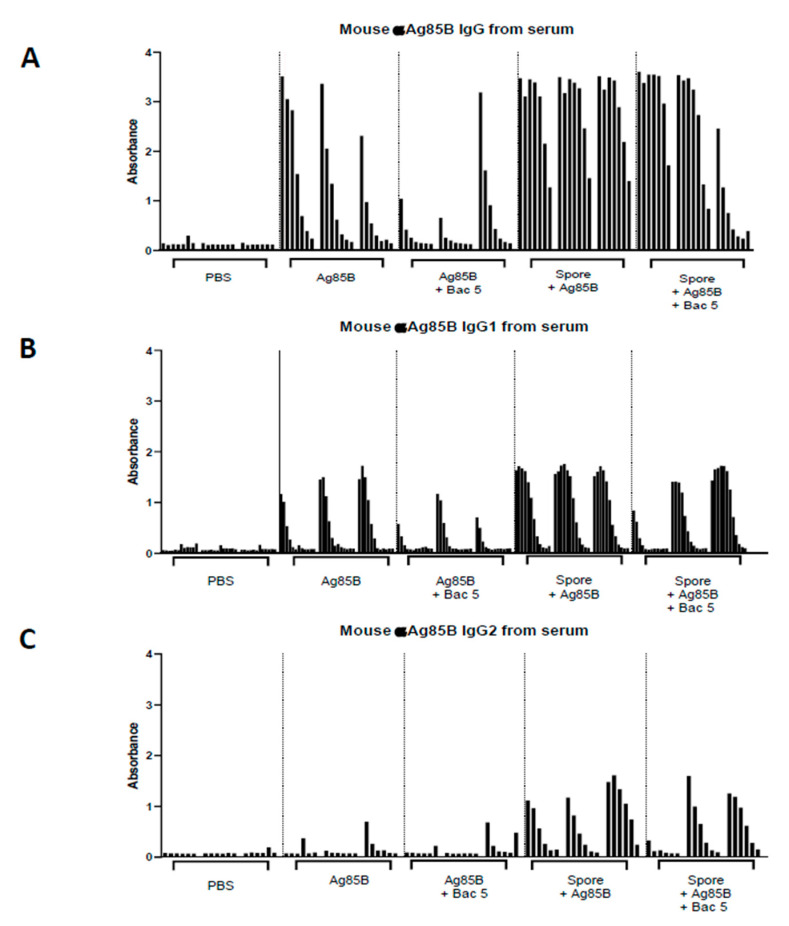
Adjuvant Effect of *B. subtilis* spores on IgG Titres in Response to Ag85 Vaccination. Eight to ten-week old female C57BL/6 mice were immunised with either PBS, Ag85B, or Ag85B plus *B. subtilis* spores, Bac5 peptide, or both combined (*x*-axis). All units (n = 3 per group) received two doses, three weeks apart and three weeks after the final dose. Serum was recovered for measurement of αAg85B-specific total IgG antibody (**A**), IgG1 (**B**) and IgG2 (**C**) subclass titres using ELISA. Sera from each biological replicate was diluted in a titration series and is displayed for each immunisation condition.

**Figure 7:**
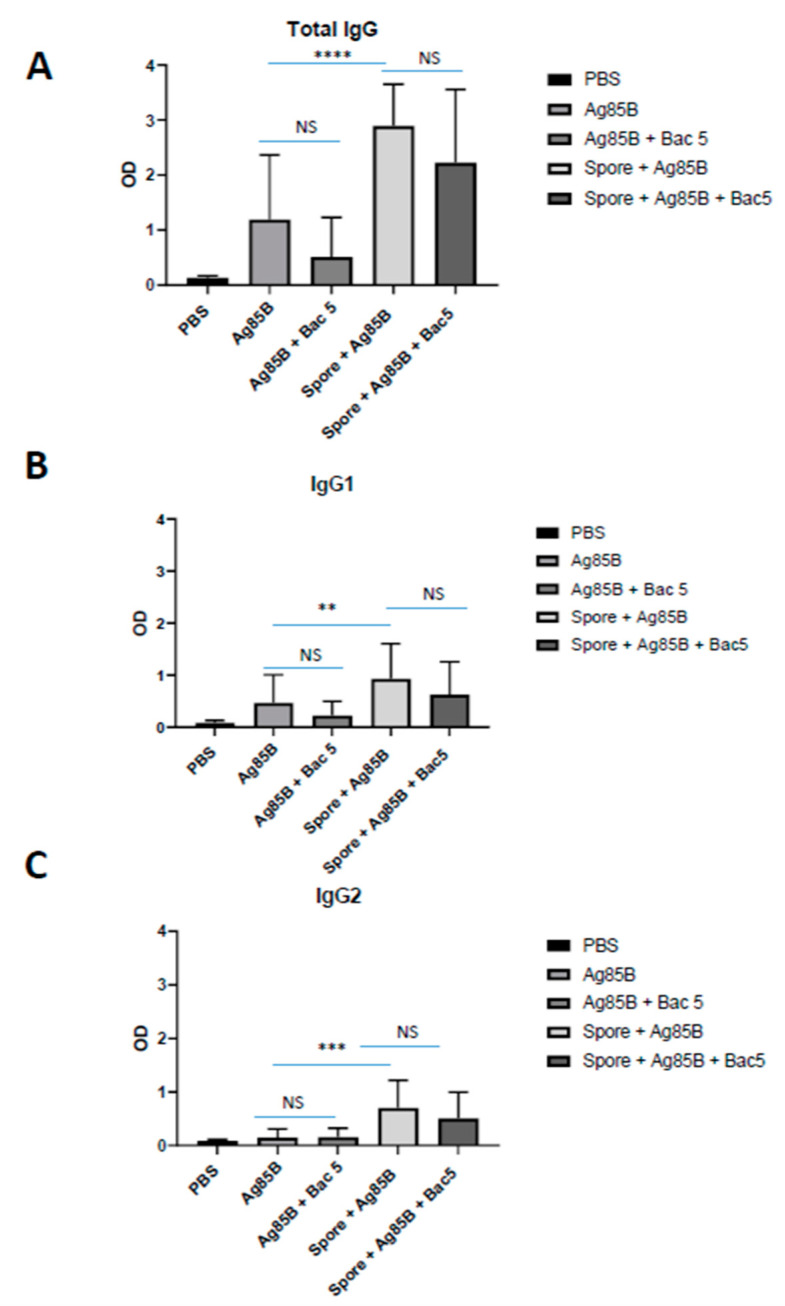
Statistical Analysis of Adjuvant Effect. The sum total optical density values from each condition, as generated by IgG ELISA, including all biological replicates (n = 3) (see Figure 6) was used to calculate the mean values with standard deviation from each immunisation condition to allow statistical comparison. Analysis was performed individually for total IgG (**A**), IgG1 (**B**) and IgG2 (**C**) subclasses. one-way ANOVA with multiple comparisons was performed between groups (**** = *p* < 0.0001, *** = *p* < 0.001, ** = *p* < 0.01, NS = No Significance).

Having studied the effect of Bac5 in murine innate immune cells in vitro, and in human tonsil cells ex vivo, we next examined the effect of the peptide in vivo using the murine model, specifically, its role in directing humoral immunity. While Bac5 appeared to be a potent stimulator of cell-mediated effectors including NO and IFNγ, it did not significantly alter antigen-specific immunoglobulin titres in the serum of vaccinated mice (Figure 6). In fact, there was a trend that Bac5 peptide reduced IgG titres in response to vaccination, but this was not statistically significant. The complete dataset shown in Figure 6 is summarised in Figure 7 with statistical comparison between mean titres from each condition. The inclusion of spores along with Ag85 was able to significantly enhance the magnitude of the specific IgG response compared with Ag85 vaccination alone, in terms of total IgG as well as both IgG1 and IgG2 subclasses.

## 3. Discussion

Antimicrobial peptides, ancient and conserved components of innate immunity, are well known for their ability to kill a broad range of pathogenic microorganisms. It has become clear in recent years that many also possess the ability to modulate host immunity. Bac5 is a bovine peptide with an unusual amino-acid sequence. Our previous work identified that besides direct killing of bacteria including *Escherichia coli* and mycobacteria, it is able to independently upregulate markers of inflammation in murine cell lines and recruit leukocytes in the zebrafish model [8]. Presently, we detail its functionality in a human ex vivo tissue model and, in particular, its role in the context of adaptive cell-mediated and humoral immunity in response to vaccination with mycobacterial Ag85. Our data suggest that Bac5 has consistent activity across different host species, supporting the validity of utilising different models to assess functionality, and highlighting the potential for non-native peptide sequences to be used in human therapeutic applications. Important to this end, Bac5 proved to have little or no cytotoxicity against the murine cell lines used in this study.

In human tissue, as in mice and zebrafish, Bac5 stimulated release of the proinflammatory effector, nitric oxide, at concentrations much lower than its antimicrobial minimum inhibitory concentration. In resting alveolar murine MH-S cells, this was not associated with a reduction in bacterial CFU when the cells were already infected with mycobacteria prior to treatment. It would be interesting to examine whether pre-treatment of the cells with Bac5 confers antimicrobial activity against subsequent infection. Bac5 was additionally capable of altering the expression of cell-surface markers independent of costimulation with PAMP. This suggests an interesting role for the peptide in altering the phenotype of bystander cells close to the foci of infection, where Bac5 is secreted by degranulating neutrophils [23]. In support of this proposed role, we previously identified that Bac5 can sensitise epithelial cells to TNFα, and it has recently been shown that certain other bovine AMP can promote sensing of nucleic acids, also in epithelial cells [24]. Specifically, we found that expression of CD86; CD40; MHCII; CCR7; CD80 and MHC I were all increased by Bac5. Human cathelicidin [25] and beta defensins [26] were similarly reported to upregulate expression of dendritic cell endocytic receptors and antigen presentation markers, and to support T_H_1-polarised cytokine secretion. This is an interesting finding, as it points to Bac5 potentially influencing the early cross-talk between antigen-presenting cells and T-cells, rather than having a role limited exclusively to the direct killing of pathogens. MHC-peptide complexes, supported by CD80 and CD86, are crucial for T-cell receptor engagement and subsequent activation of T-helper cells, which in turn impact B-cell and macrophage responses. When expressed and activated on dendritic cells, CCR7 has been described to mediate trafficking to secondary lymphatic centres, where training of naive T_H_0 T-cells occurs [27]. We did not examine CCR7 expression by T-cells in this context, but its activation has been described to enhance IFNγ production and T_H_1 T-cell proliferation [27].

When Bac5 was coupled with PAMP in the form of *B. subtilis* spore-adsorbed Ag85, it significantly enhanced IFNγ production in human ex vivo isolated tonsil cells. This effect was only observed when the peptide was combined with Ag85 and was not apparent when either Bac5 alone, or spore-Ag85 without Bac5 was used. This is interesting considering an additive effect of Bac5 coupled with PAMP was not observed for either nitric oxide (MH-S cells) or TNFα (tonsil cells) secretion. We previously reported a similar effect whereby human THP-1 cells optimally synthesised IL-1b when costimulated with both Bac5 and *M. marinum* in combination [8]. The upregulation of IFNγ is highly relevant to *M. tuberculosis* infection, in which it has been demonstrated to be a crucial innate immune effector in controlling disease progression [28].

Since the same conditions did not induce IFNγ production in either CD4^+^ or CD8^+^ T-cells, additional cell types in the whole tonsil cell population may be responsible for the observed IFNγ release. Although activated T-cells and natural killer cells are regarded as the major source of IFNγ during early infection, macrophages are also capable of secreting this cytokine in the presence of IL-12 and IL-18 [29]. We did not find upregulation of IFNγ in Bac-5-treated MH-S cells. However, this experiment used murine rather than human cells, and Bac5 was not tested in combination with spore-adsorbed Ag85. Combined with the ability of Bac5 to recruit macrophages and neutrophils, the stimulation of IFNγ production from alveolar macrophage-like cells suggests a potential therapeutic application for Bac5 in the treatment of tuberculosis.

Spore-adsorbed Ag85 was able to significantly increase the magnitude of Ag85B-specific IgG titre compared with Ag85 alone. This effect was observed in total IgG as well as IgG1 and IgG2 subclasses, and supports our previously published finding that *B. subtilis* spores, which induced a mixed T-cell cytokine profile, supported humoral immunity upon vaccination with mycobacterial antigens [15]. Besides targeting of mucosal tissue, this adjuvancy is likely to be supported by the triggering of pattern recognition receptors by spore antigens and, potentially, by a depot effect whereby the spores retain the mycobacterial antigens at the injection site.

By contrast, Bac5 peptide failed to demonstrate an adjuvant effect, either alone or when adsorbed onto the spore surface alongside vaccination with Ag85B. We considered whether adsorption of the peptide to the spore may either degrade the spore or mask adjuvenic epitopes. We confirmed by flow cytometry that FITC-labelled Bac5 was associated with the spores, and there was no change in the forward or side scatter of the spores, suggesting no physical degradation. Previously, we showed that the adjuvenic effect of the spores was not affected by spore viability [15]. Additionally, the data show that the spores remain potent stimulators of the IgG response even with surface-adsorbed Bac5 since higher titres were observed in this condition than with Bac5 alone.

Peptide-based adjuvants have been explored previously, and some have demonstrated the ability to enhance the expression of protective immunological markers and improve the efficacy of certain vaccines, including against *M. tuberculosis* [12,30]. Recently, a plant-derived polysaccharide adjuvant, Advax, that induces responses similar to Bac5, such as leukocyte recruitment and T_H_1 inflammation, improved protection against *M. tuberculosis* challenge [31]. While the concept holds promise, our data suggest that despite being able to recruit and activate macrophages, Bac5 is not a suitable adjuvant candidate, possibly due to the significant bias it induces towards a T_H_1 type outcome. For instance, IFNγ and CD86, which are upregulated by Bac5, both support T_H_1 polarisation of CD4^+^ T-cells via enhanced IL-12 production by antigen-presenting cells [32], while nitric oxide can suppress the proliferation and function of T_H_17 cells, which contribute to expansion of antigen specific B-cells and enhanced antibody titres [33]. It is likely that a more balanced outcome that additionally engages aspects of T_H_2 and T_H_17 immunity, linked with development of immune memory in mucosal tissue, is desirable [20].

In conclusion, our data support a prominent, immunomodulatory role for Bac5 as a conductor of the early, cell-mediated immune response, capable of activating noninfected cells, enhancing IFNγ secretion alongside PAMP and altering surface marker expression in alveolar macrophage-like cells above and beyond its additional role as a directly antimicrobial peptide.

## 4. Materials and Methods

### 4.1. Peptide Information

Bac5 and Bac5-FITC were synthesised by Biomatik Corporation, Ontario, Canada. We used the active portion of the Bac5 sequence (Bac5 = RFRPPIRRPPIRPPFYPPFRPPIRPPIFPPIRPPFRPPLGPFP), representing the cleaved product from the inactive precursor sequence (UniProt entry: CTHL2_BOVIN). The peptides were prepared as acetate salt formulations, with >99% TFA removal and purity assessed by HPLC.

### 4.2. Bacterial Growth

Mycobacterial strains (*M. bovis* BCG and *M. tuberculosis* H37Rv) were grown in Middlebrook 7H9 media (containing 0.2% glycerol, 10% ADC, Selectatab). All cultures were incubated at 37 °C until log phase (OD_600_ = 1.0–1.2).

### 4.3. Cell Culture

Murine alveolar macrophage MH-S cells were grown in 75 cm^2^ tissue culture flasks (Corning) in complete RPMI-1640 (Sigma-Aldrich Company Ltd, Gillingham, UK) supplemented with 2 mM L Glutamine, 0.05 mM 2-mercaptoethanol, 10 U/mL Penicillin, 50 μg/mL Streptomycin and 10% (*v*/*v*) FBS, and maintained at 37 °C, 95% RH and 5% CO_2_.

### 4.4. Haemolytic Assay

Fresh blood was obtained from St Georges Hospital blood testing department. Blood was centrifuged at 1000× *g* for 5 min. The red blood cell (RBC) pellet was mixed with equal volume of 1X phosphate buffered saline (PBS) (pH 7.4) before recentrifugation. 140 µL of 5.14% RBC solution was added to the first row of a 96 well plate (Corning). 90 µL 4% RBC solution was subsequently added to all the other wells. 40 µL of Bac5 was then added to the top row and serially diluted two-fold across the plate to get a concentration range of 100 to 0.78 µg/mL in triplicates. PBS and RBC only were used as negative controls, while 0.2% Triton-X was used as a positive control. The plate was incubated for 1 h at 37 °C. It was then centrifuged at 1000× *g* for 5 min at 4 °C. Following the formation of RBC pellets, 70 µL of supernatant was removed from each well and transferred into clear, flat, round bottom 96 well plate (Nunc), ensuring no pellets were disturbed. The absorbance was read using TECAN Sunrise spectrophotometer at 450 nm.

### 4.5. Cytotoxicity Assay

The cytotoxicity assay was performed in a 96-well plate based on Resazurin Microtitre assay (REMA) as described previously [34,35]. Briefly, Bac5 was serially diluted (two-fold) with RPMI/FBS media in a final volume of 100 µL in 96-well plate to achieve concentrations of 100–0.78 µg/mL in triplicates. In each well, 100 µL MH-S cells or NHP lymphocytes (5 × 10^5^ cells per mL) were added and the plates were incubated in a CO_2_ incubator at 37 °C. Following a 48-h incubation, 30 µL 0.01% freshly prepared resazurin solution was added to each well and plates were further incubated overnight in the CO_2_ incubator. The absorbance was then read at 590 nm using TECAN Sunrise spectrophotometer.

### 4.6. Bac5 Binding to Spores and MHS

To determine the binding of Bac 5 to spores, a FITC conjugated Bac 5 was generated. Dead and live spores were prepared according to Copland et al. [15]. For the binding assay, suspensions containing 2 × 10^9^ spores were centrifuged and pellets were suspended in 0.2 mL of 1X PBS (pH 7.4) in 1.0 mL tubes. Bac5 conjugated to FITC at 1, 10, 25, 50 µg/mL were added to the spore suspensions, and the binding mixture was incubated for 1 h at room temperature. Spore only was taken as spores were centrifuged at 400× *g* for 5 min, and the pellet was washed twice with 1X PBS. Finally, 200 µL of spore suspension was added to 1X FACS buffer (1X PBS, 0.5% BSA, 0.01% Na Azide). A further 1/20 dilution was carried out using 1X FACS buffer for each tube to limit the number of spores to 5 × 10^6^ spores/mL. Binding of Bac5 to spores was evaluated by flow cytometry using a BD FACSCalibur on the basis of their scatter/fluorescence characteristics and the presence of FITC signal.

Similarly, for evaluating binding of Bac5 to MH-S cells, two-fold serial dilutions of FITC conjugated Bac5 were performed between 100 mg/L and 0.15 mg/L (in triplicates) with phenol-free RPMI media (Sigma) in 96-well U-bottom plates (Corning). A 100 µL of MH-S cells (5 × 10^5^ cells/mL) prepared in phenol-free RPMI/FBS media, was added to the plate. The plate was incubated for at 37 °C for 24 h. After incubation, the cells were centrifuged at 400× *g* for 5 min, and washed twice in 1X PBS. The cells were then resuspended in 200 µL of 1X FACS buffer. The cells were analysed by flow cytometry and FITC signal intensity measured. To confirm the viability of MH-S cells in presence of Bac5, an identical plate as above was prepared and incubated at 37 °C for 24 h. After incubation and washes in 1X PBS, the cells in each well were resuspended in 50 µL of eFluor780 dye (Invitrogen). The plate was incubated at 4 °C for 15 min. The cells were then resuspended in 200 µL of 1X FACS buffer, washed twice and then analysed by flow cytometry.

### 4.7. Nitric Oxide Assay

Serial dilutions of Bac5 were made between 50 mg/L and 0.39 mg/L (in triplicates) with phenol-free RPMI media (Sigma) in 96-well U-bottom plates (Corning, NY, USA). 1 × 10^5^ MH-S cells were added to all wells. *M. bovis* BCG (grown to log phase OD600 = 1.0) was added at a multiplicity of infection (MOI) of 1. Plates were sealed and incubated in a CO_2_ incubator at 37 °C for 18 h. The plate also contained MH-S cells, *M. bovis* BCG and media- only controls. Post incubation, 100 µL of sample from each well was mixed with 100 µL of Griess reagent and incubated in the dark at room temperature for 6 h. Griess reagent was prepared as per manufacturer’s instructions (Sigma-Aldrich). The absorbance was then read using TECAN Sunrise spectrophotometer at 560 nm.

### 4.8. Intracellular Survival Assay

The in vitro infection assay was carried out in a Category III laboratory. To set up the infection model, MH-S cells were centrifuged at 1200× *g* for 10 min and resuspended in fresh RPMI/FBS media and 5 × 10^5^ cells/mL were then added to a 24-well plate. To this, Bac5 at concentrations of 100, 50, 25, 1 µg/mL were added in duplicates, and the plates were incubated at 37 °C, 5% CO_2_ for 16 h. After 16 h, 1 × 10^6^ cells/mL of *M. tuberculosis* H37Rv prepared in RPMI/FBS were added to the wells to achieve a MOI of 2. The plates were incubated at 37 °C, 5% CO_2_ for 4 h. After incubation, the cells were centrifuged at 1200× *g* for 10 min, and resuspended in fresh RPMI/FBS media. 200 µg/mL of amikacin was added to test wells to kill extracellular bacteria, and the plate was incubated further for 2 h at 37 °C, 5% CO_2_. Post incubation, the cells were lysed by adding 0.1% Triton-X and centrifuged at 16,000× *g* for 10 min at RT. The pellet was resuspended in 200 µL of Middlebrook 7H9 media and two dilutions were plated on Middlebrook 7H11 agar containing 10% (*v*/*v*) of Middlebrook Oleic Albumin Dextrose Catalase Growth Supplement (OADC). The plate was incubated at 37 °C for 14 days to determine intracellular survival of *M. tuberculosis*.

### 4.9. MHS Cell-Surface Marker Expression

MH-S cells were used at a density of 1 × 10^6^/mL and stimulated with Bac 5 at 10 μg/mL or 100 μg/mL for 72 h in triplicate wells. Cells were then stained for 10 min at 4 °C with the viability dye, eFluor_780. Cells were then washed and stained with anti-mouse CD40_FITC; MHC II_PE; CD86_APC; PDL1_PE Cy7; CCR7_PerCP Cy5.5; MHC I_BV421; CD80_BV510 (all sourced from: Biolegend UK Ltd, London, UK) for 30 min at 4 °C. Cells were washed and resuspended in FACS buffer. Cell-surface marker expression was assessed using a Beckton Dickinson Canto II flow cytometer. Cells were gated by size and doublets and nonviable cells were excluded. Flow cytometry analysis was performed using FlowJo software version 10.6.2, with 30,000 events recorded for each condition.

### 4.10. Human Tonsil Mononuclear Cell Culture

Human tonsil tissue used in this study was obtained as part of a clinical study entitled “Vaccine immunogenicity using tuberculosis and dengue fever as models” (IRAS project ID 239627). This study is run by Prof Reljic’s group in collaboration with the ENT department at St Georges Hospital NHS Trust. Ethical approval for this project was provided by the SGUL JREO and the South Central Oxford-C NRES Committee.

Tonsil mononuclear cells (TMCs) were generated from tonsil tissue and used to determine the immunogenicity of vaccine candidates. To prepare TMCs, tonsil tissue was aseptically cut into fragments using a sterile scalpel. Cells were passed through a 70 µM strainer using R1O media and the sterile plunger of a 10 mL pipette. Cells were then suspended in 50 mL R1O media and 20 mL gently pipetted on to 20 mL Ficoll (density of 1.077 g/mL, GE Healthcare) and the cells spun for 30 min at 700× *g* without brake. The supernatant was removed and cells resuspended in R1O media. Cells were counted and cell viability was determined using trypan blue. Cells were spun at 400× *g* for 10 min and the supernatant discarded. Frozen stocks were prepared by resuspending cells in freezing media at a concentration of 1 × 10^7^ cells/mL. Cells were frozen at −80 °C and then stored in liquid nitrogen.

### 4.11. IFNγ and TNFα ELISA

ELISAs were used to determine the production of IFNy and TNFα by stimulated tonsil cells. Cells were used at a density of 1 × 10^6^ cells per mL in 96-well plates, with 1 × 10^5^ cells in each well, cultured in RPMI 1640 media at 37 °C. They were stimulated with either Bac 5 (10 ng/mL), *B. subtilis* spores (100:1 ratio to cells) or Ag85B (5 μg/mL) and supernatant collected for ELISA. Human TNFα or Human IFNγ uncoated ELISA kits from Invitrogen were used according to the manufacturer’s instructions. Anti-TNFα or Anti IFNy capture antibody was diluted 1/250 in 1× coating buffer. 100 µL were incubated overnight in wells of a 96-well ELISA plate (Nunc, Maxisorp). The plate was washed three times with PBS with 0.05% Tween. Wells were blocked with assay diluent for 1 h and then washed. Cytokine standards were prepared in 1× assay diluent and 100 µL added to wells. Supernatant from tonsil cultures was added to wells and then serially diluted 1 in 2 in assay diluent. These plates were incubated for 2 h and then washed. 100 µL of Anti TNFα or Anti IFNy detection antibody diluted 1/250 in assay diluent was added to each well. The plates were incubated for 1 h at room temperature and subsequently washed. 100 µL of Avidin HRP diluted 1/250 in assay diluent was added. The plate was incubated for 30 min and washed five times. 100 µL of TMB substrate solution was added. After 1 min, the colour developed and 100 µL of 0.2 M sulphuric acid was added to wells. The plate was then read in a TECAN Sunrise absorbance microplate reader at 450 nm.

### 4.12. TMC Intracellular Cytokine Assay

This assay established the direct stimulatory effect of different vaccine formulations on the production of IL-2, IFNy and TNFα by T-cells. This assay also investigated the proliferation of CD4^+^ and CD8^+^ T cells through intracellular expression of Ki67. Tonsil mononuclear cells were used at a density of 1 × 10^6^ cells per mL in 96 well plates, with 1 × 10^5^ cells in each well. Cells were cultured in supplemented RPMI 1640 media grown at 37 °C, 5% CO_2_, 95% humidity. A range of vaccine formulations were tested. Bac 5 was added at either 10 ng/mL, 100 ng/mL or 1 μg/mL. *B. subtilis* spores were added at a ratio of 100:1 to cells. Ag85B was added at a concentration of 5 μg/mL.

Cells were washed lysed with permeabilization buffer consisting of FACs buffer with 0.5% saponin. Intracellular staining was performed on the cells for 45 min at 4 °C with the following antibodies; CD3_FITC (Biolegend), CD4_PerCP Cy5.5 (Biolegend), CD8_BV510 (Biolegend), IL-2_PE (Biolegend), IFNy_BV421 (Biolegend), TNFα_PE-Cy7 (Biolegend) and Ki67_APC (Biolegend). Expression levels of CD3, CD4, CD8, Ki67, IL-2, TNFα, and IFNy were measured by flow cytometry. Cells were gated on size and linearity. Nonviable cells were excluded. T cells were gated by expression of CD3 and then CD4 and CD8. Fluorescence minus one (FMOs) were used to set the appropriate gates and determine positive populations.

### 4.13. In Vivo Murine Studies

All animals were used with approval from St. George’s University of London Ethics Committee under an approved UK Home Office animal project license and used in accordance with the Animals (Scientific Procedures) Act 1986. Eight- to ten-week-old female C57BL/6 mice were used for this study obtained from Charles River, Kent, UK. Animal work was conducted at St. George’s University of London Biological Research Facility in accordance with local guidelines, including approval from the St. George’s University of London Research Ethics Committee and national legislation, the Animals in Scientific Procedures Act, 1986. All procedures were performed under the approved UK Home Office animal project license.

C57BL/6 mice were divided into five groups, each containing three mice. The mice were immunised with; PBS only, Ag85B only, Ag85B and Bac 5, Ag85B and spores or Ag85B with spores and Bac5. 50 µg of Ag85B and Bac 5 was administered per dose, whilst 1 × 10^9^ spores were in each dose. Animals received two doses, three weeks apart. A further three weeks after the second dose, the animals were culled, and blood was collected for quantification of anti-Ag85B IgG in serum by ELISA.

### 4.14. αAg85B-Specific IgG ELISA

96 well ELISA plates (Nunc, Maxisorp) were coated with 100 µL of 5 µg/mL Ag85B (Lionex GmbH, Braunschweig, Germany) in coating buffer overnight. The plate was washed three times with 1 × PBS with 0.05% Tween 20. Wells were then blocked with assay diluent (eBioscience, Thermo Fisher Scientific, UK) and incubated for 1 h at RT. The plate was then washed three times. Serum was serially diluted 1:2 in assay diluent and transferred to 96 well plate precoated with Ag85B and incubated at RT for 2 h. The plate was then washed five times. To detect total IgG, α-mouse IgG (Fc Specific) conjugated to horseradish peroxidase (Sigma, UK) was used. Anti-Ag85B IgG1 was detected using α-mouse IgG1 (γ chain specific) conjugated to horseradish peroxidase (Sigma). These detection antibodies were diluted 1/250 final concentration in assay diluent and 100 µL added to each well and incubated for 1 h at RT and then washed five times. 100 µL substrate solution was added and after 1 min, the colour developed and 100 µL of 0.2 M sulphuric acid was added to all wells. The plate was then read in a TECAN Sunrise absorbance microplate reader at 450 nm.

## Supplementary Materials

The following are available online at https://www.mdpi.com/2079-6382/9/12/926/s1, Figure S1: Bac5 Readily Binds to MH-S Cells and is not Cytotoxic. Figure S2. FITC-Bac5 binding to bacterial spores.
