# Supplementary material for: The Antimicrobial Peptide, Bactenecin 5, Supports Cell-Mediated but Not Humoral Immunity in the Context of a Mycobacterial Antigen Vaccine Model
Source: Antibiotics (Basel). 2020 Dec 19;9(12):926. doi: 10.3390/antibiotics9120926 (PMC7766334; doi:10.3390/antibiotics9120926)
Supplement: Supplementary file 1 [file antibiotics-09-00926-s001.pdf]

A

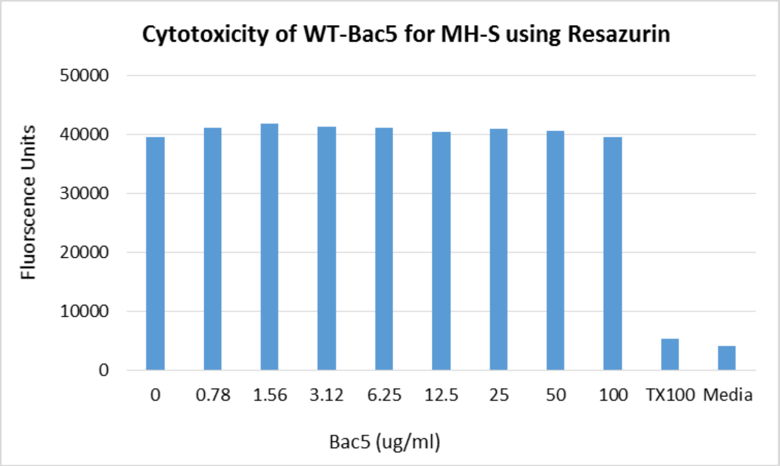
Cytotoxicity of WT-Bac5 for MH-S using Resazurin
Fluorscence Units
50000
40000
30000
20000
10000
0
0
0.78
1.56
3.12
6.25
12.5
25
50
100
TX100
Media
Bac5 (ug/ml)

C

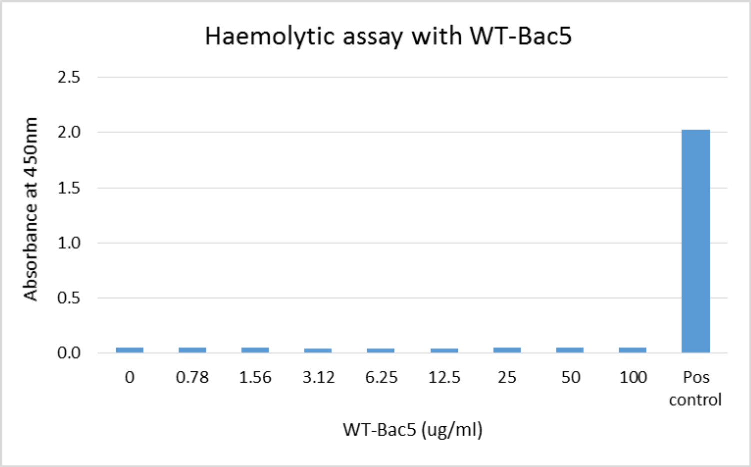
Haemolytic assay with WT-Bac5
Absorbance at 450nm
2.5
2.0
1.5
1.0
0.5
0.0
0
0.78
1.56
3.12
6.25
12.5
25
50
100
Pos control
WT-Bac5 (ug/ml)

B

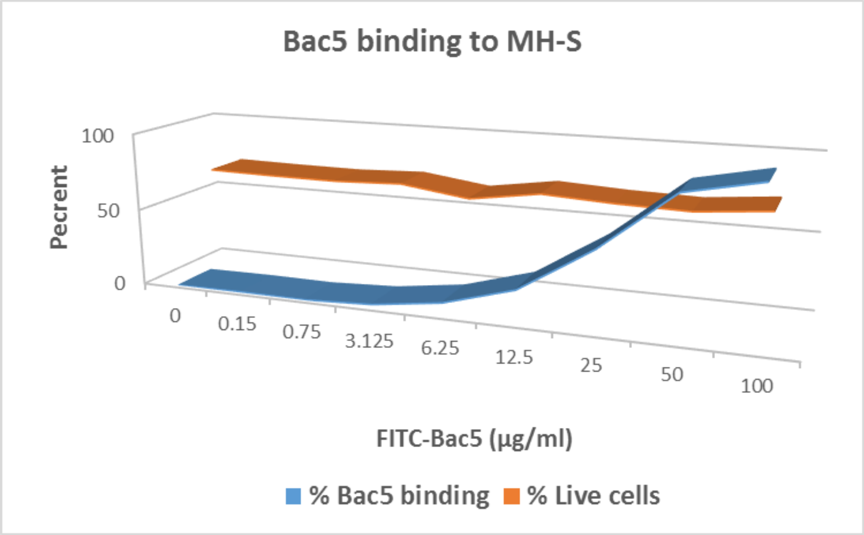
Bac5 binding to MH-S
Pecrent
100
50
0
0
0.15
0.75
3.125
6.25
12.5
25
50
100
FITC-Bac5 (μg/ml)
% Bac5 binding
% Live cells

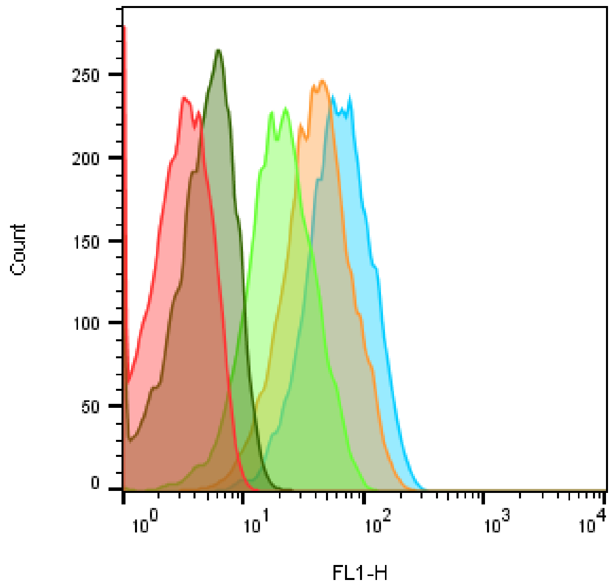

	SampleID	Geometric Mean : FL1-H
	Control (no peptide)	2.88
	Peptide (1ug/ml)	4.51
	Peptide (10ug/ml)	19.7
	Peptide (25ug/ml)	39.5
	Peptide (50ug/ml)	60.0

Live spores

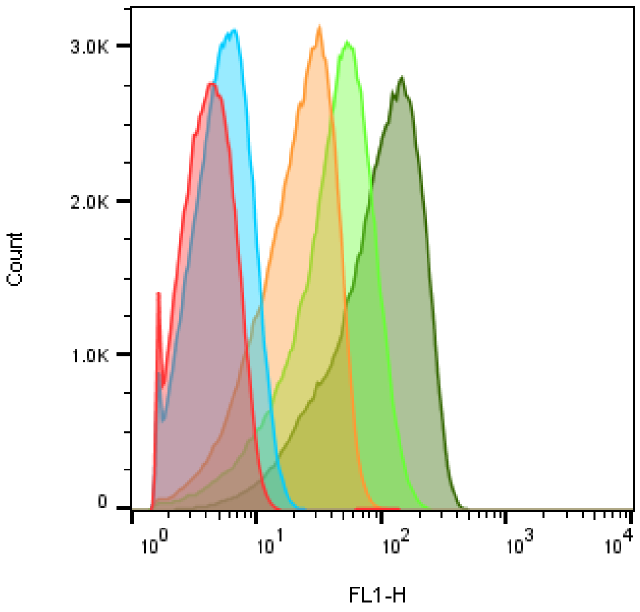

	SampleID	Geometric Mean : FL1-H
	Control (no peptide)	4.13
	Peptide (1ug/ml)	5.21
	Peptide (10ug/ml)	19.6
	Peptide (25ug/ml)	37.6
	Peptide (50ug/ml)	85.1

Dead spores
